# Increasing Importance of Clinical Nutrition for Arterial Health

**DOI:** 10.3390/nu14081532

**Published:** 2022-04-07

**Authors:** Stefan Acosta

**Affiliations:** 1 Department of Clinical Sciences, Lund University, 21421 Malmö, Sweden; stefan.acosta@med.lu.se; 2 Vascular Center, Department of Cardiothoracic and Vascular Surgery, Skåne University Hospital, 20501 Malmö, Sweden

## 1. Modifiable Risk Factors beyond Smoking

Cardiovascular disease (CVD) is the leading cause of death in high-income countries. A healthy lifestyle can to some extent counteract a genetic predisposition to CVD [1]. Indeed, studies on modifiable risk factors for cardiovascular disease have become increasingly important. The declining prevalence of smoking in high-income countries [2] will lead to a greater importance of the other main modifiable risk factors for arterial health such as diet, alcohol, weight and physical activity. This Special Issue, entitled “Clinical nutrition for arterial health”, includes papers emphasizing the role of nutrition for sustained arterial health.

Four prospective cohort studies from high-income countries were collected: two of them had a cross-sectional [3,4] design using ultrasound characteristics of subclinical atherosclerosis as an endpoint, while two had a longitudinal [5,6] design using inpatient registries for clinical atherosclerotic disease as an endpoint. All studies used validated questionnaires to capture intake of food and beverages.

## 2. The Cross-Sectional Studies

After adjusting for methodological, lifestyle and dietary confounders, the authors found no linear association between sugar intake and intima media thickness in the report examining healthy participants [4]. Patients referred to the outpatient clinic at the Department of Internal Medicine (section Cardiology and Angiology) with routine indications for ultrasound examinations of the carotid and/or femoral arteries were screened, and patients with at least one risk factor for CVD were eligible for inclusion [3]. Total alcohol consumption increased the risk, and consumption of vegetables decreased the risk, for total plaque volume in the multivariate prediction model.

## 3. The Longitudinal Studies

Participants without ischemic stroke and atrial fibrillation/flutter were followed for 21.2 years, and 2339 individuals were diagnosed with atherothrombotic ischemic stroke unrelated to atrial fibrillation or flutter out of 26,547 participants [5]. The diagnosis of ischemic stroke was confirmed in 89% of a random sample. Non-smoking status, high diet quality and high physical activity in leisure time were associated with 38%, 17%, and 11% decreased risk, respectively, of incident atherothrombotic ischemic stroke, independently of established risk factors, with non-significant associations with body mass index and alcohol intake. The effect of the lifestyle factors was independent of predisposing comorbidities at baseline.

Participants without cardiovascular disease and atrial fibrillation or flutter were followed for 21.1 years, and 5858 individuals were diagnosed with any atherosclerotic cardiovascular disease unrelated to atrial fibrillation or flutter out of 26,990 participants [6]. The diagnosis of coronary artery disease, ischemic stroke, peripheral arterial disease and carotid artery disease were confirmed in 97%, 89%, 98% and 99%, respectively. Showing the benefits of higher diet quality, intake of fish, shellfish, fiber and saturated fatty acid was associated with 6%, 5%, 7% and 4% decreased risk for incident atherosclerotic cardiovascular disease. High physical activity during leisure time was associated with an 18% decreased risk and obesity with a 17% increased risk of incident atherosclerotic cardiovascular disease. The presence of diabetes mellitus and current smoking at baseline were the two risk factors with the highest risk for incident atherosclerotic cardiovascular disease (Hazard Ratios of 2.26 and 2.24, respectively).

## 4. Call for New Longitudinal Cohort Studies

Middle-aged participants in three of the studies in this Special Issue were recruited between 1991 and 1996. Timing was perfect to evaluate risk factors at baseline for development of cardiovascular disease in this cohort. However, major changes in risk factor expression at baseline, diagnosis and treatment of cardiovascular disease have occurred. While the prevalence of smoking has decreased substantially in high-income countries, the prevalence of obesity and diabetes mellitus have been increasing in low-, middle- and high-income countries [7]. The expression of cardiovascular burden is expected to change in the future. Dietary factors are highly likely to have a greater impact on the development of CVD. The establishment of prospective cohort studies with collection of contemporary baseline data [8] is therefore warranted.

## 5. Future Directions and Challenges

The link between sugar intake and cardiovascular disease needs to be evaluated in a randomized trial with a long-term follow up [9]. It is also important that such trials are independent of industry funding. Future prospective longitudinal cohort studies should include repeated assessments of semi-modifiable and modifiable risk factors, and subclinical and clinical atherosclerosis, to better estimate the relation between clinical nutrition and cardiovascular disease.
